# A Case of Anti-Ma2 Encephalitis Presenting with Pendular Torsional Nystagmus

**DOI:** 10.1007/s12311-023-01601-w

**Published:** 2023-10-18

**Authors:** Gloria Vaghi, Elisa Vegezzi, Paola Bini, Matteo Gastaldi, Luca Diamanti, Enrico Marchioni, Silvia Colnaghi

**Affiliations:** 1 Department of Brain and Behavioral Sciences, University of Pavia, Pavia, Italy; 2 Headache Science and Neurorehabilitation Center, IRCCS Mondino Foundation, Pavia, Italy; 3 Neuroncology and Neuroinflammation Unit, IRCCS Mondino Foundation, Pavia, Italy; 4 Neuroimmunology Research Unit, IRCCS Mondino Foundation, Pavia, Italy; 5 Neurology Unit, Dipartimento Funzionale Sperimentale Interaziendale di Neuroscienze, ASST, Pavia, Italy

**Keywords:** Paraneoplastic neurological syndrome, Anti-Ma2 encephalitis, Nystagmus, Immune checkpoint, Anti-PD-L1

## Abstract

Antibodies against the neuronal protein Ma2 have been reported in a peculiar form of paraneoplastic encephalitis with prominent involvement of the limbic, brainstem, and diencephalic structures and usually associated with germ cell testicular, lung, or breast cancer. The diagnosis is frequently challenged by atypical clinical manifestations including parkinsonism, sleep disturbances, hypothalamic-pituitary dysfunctions, and motor neuron-like syndrome. In recent years, the advent of monoclonal antibodies targeting immune checkpoints has deeply changed the treatment of different tumors, especially melanoma and lung cancer. However, given their nature, an increasing number of neurological immune-related adverse events, including ocular motor abnormalities, have been described. Here, we report a woman with advanced non-small cell lung cancer treated with anti-PD-L1 durvalumab, presenting with an isolated pendular torsional nystagmus, in association with anti-Ma2 antibodies. This peculiar case widens our knowledge on the clinical presentation of anti-Ma2 encephalitis associated with checkpoint inhibitors.

**Supplementary Information:**

The online version contains supplementary material available at 10.1007/s12311-023-01601-w.

## Introduction

Paraneoplastic neurological syndromes (PNSs) are typically associated with an underlying tumor. The finding of anti-neuronal or glial autoantibodies supports an immune-mediated cause of PNSs and provides a useful clinical biomarker. Both the type of clinical syndrome and autoantibody is graded from low to high risk in regard to the likelihood of the presence of an underlying tumor. In addition, specific autoantibodies can associate with a peculiar clinical syndrome and/or, more commonly, with a certain type of tumor [1].

Among PNSs, anti-Ma2 encephalitis typically presents with prominent involvement of the limbic, brainstem, and diencephalic structures, usually in association with germ cell testicular, lung, or breast cancer [2, 3]. The diagnosis can be challenged by atypical clinical manifestations including parkinsonism, sleep disturbances, weight gain, sexual dysfunction, and motor neuron-like disease [3]. Changes on brain magnetic resonance imaging and inflammatory markers in the cerebrospinal fluid may support the diagnosis [3].

Recently, the use of immune checkpoint inhibitors (ICIs), monoclonal antibodies targeting immune checkpoints, has deeply affected the treatment of different tumors, especially melanoma and lung cancer. ICIs act by enhancing the host immune response against the tumor through the blockage of immune checkpoints (including cytotoxic T lymphocyte–associated antigen 4 (CTLA-4), the programmed death-1 receptor (PD-1), and its ligand (PD-L1)). However, such a mechanism can be associated with immune-related adverse events that can target any organ, including the nervous system (n-irAE). Neurological adverse events can take different forms, potentially involving peripheral and central nervous system, e.g., Guillain-Barré-like syndrome, other peripheral neuropathies, or encephalitis, to name a few [4]. The differential diagnosis between PNS and PNS associated with ICIs is often challenging because of the shared clinical presentations and biohumoral findings. Nonetheless, a clinical onset soon after ICI initiation, usually within 4 months after starting treatment, as well as the lack of a close temporal relationship with tumor diagnosis, supports an ICIs-related PNS [4, 5].

As previously reported, anti-Ma2 encephalitis can be associated with ICIs [4, 6], although the underlying mechanism is still unknown.

In anti-Ma2 encephalitis, several ocular motor abnormalities have been reported so far, such as ocular flutter and opsoclonus [7], horizontal or vertical nystagmus, skew deviation, vertical gaze palsy, and partial to complete external ophthalmoplegia [3, 8]. Occasionally, eye movement alterations are the only initial symptoms [3].

Ocular motor abnormalities have also been rarely described during treatment with ICIs [9–11], including vertical or horizontal nystagmus associated with acute cerebellar ataxia or brainstem encephalitis.

Here, we report on a case of a woman diagnosed with advanced non-small cell lung cancer (NSCLC) and started on anti-PD-L1 durvalumab who developed an isolated pendular torsional nystagmus and tested positive for anti-Ma2 antibodies. To our knowledge, this is the first description of this peculiar nystagmus in a patient who tested positive for anti-Ma2 antibodies.

## Case Presentation

A 71-year-old woman with uneventful clinical history developed a dry cough, dyspnea on exertion, and occasional hemoptysis in late August 2020.

Chest CT scan revealed a bulky mass localized in the perihilar area of the left upper lung with vascular and mediastinal invasion and one ileo-mediastinal lymphadenopathy on the same side. Positron emission tomography scan excluded distant metastases. The tumor was graded as IIIb, T4N2M0. Soon after, a needle biopsy disclosed a NSCLC with a Tumor Proportion Score (TPS) of programmed death ligand-1 (PD-L1) equal to 25%.

From November 2020 until April 2021, she underwent and failed multiple chemotherapy regimens and radiation.

In April 2021, given only a partial response and her clinical condition (Eastern Cooperative Oncology Group Performance Status = 1), she was started on the anti-PD-L1 durvalumab at 10 mg/kg every 2 weeks.

Since the summer 2021, she became bedridden from a disabling low back pain unresponsive to common painkillers. Magnetic Resonance Imaging (MRI) identified pelvic bone metastases.

At the end of July 2021, after the seventh durvalumab administration, she suddenly developed oscillopsia and imbalance, which progressively worsened over the course of three weeks. She described her visual problems as a sudden appearance of an illusion of rhythmic vertical oscillation of the surrounding environment. This was associated with subjective feeling of imbalance, partly due to the difficulty in fixation. However, she did not report any falls at home, and gait was still possible without aids. The sensation disappeared with closed eyes. Durvalumab was discontinued, and because of persisting symptoms, she was hospitalized in August 2021.

**Fig. 1:**
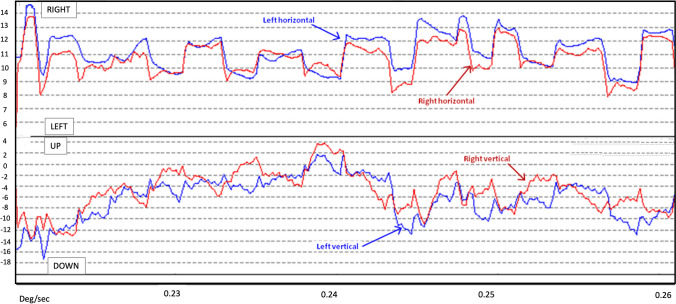
Eye position recording with Frenzel Video Goggles while the patient is fixating a target in primary position of gaze. The figure shows the position of the two eyes (red line: right eye; blue line: left eye) in the horizontal (upper panel) and vertical (lower panel) plane. Note the opposite movement of the eyes in the vertical plane (arrows)

The neurological examination showed a pendular torsional nystagmus. The torsional component was conjugate, with superimposed irregular vertical disconjugate moves (Videos and Fig. 1). The oscillation persisted under closed lids. It was inhibited in the upward gaze, while no differences in the torsional or vertical components were evident in the lateral gaze. She had difficulties in maintaining the lateral gaze position, without gaze evoked or rebound nystagmus. The neuro-ophthalmological (visual acuity, pupils, perimetry, visual evoked potentials) and neurological examinations were otherwise unremarkable. She did not present cognitive or behavioral alterations, as well as parkinsonism. Neurological examination also showed normal finger-to-nose and heel-to-shin tests and no truncal ataxia. No palatal tremor was recorded. Romberg test resulted negative. Gait was cautious, but otherwise normal.

Brain contrast MRI was normal; in particular, no olivary hypertrophy was evident. Cerebrospinal fluid (CSF) analysis showed CSF restricted oligoclonal bands. No tumoral cells were detected at CSF cytology.

The patient underwent a comprehensive laboratory examination for suspected PNS. Anti-Ma2 antibodies were detected on both serum and CSF by indirect immunofluorescence on monkey cerebellum as screening (which showed a typical neuronal nucleolar staining pattern; Euroimmun, Lubeck), and their positivity was further confirmed by commercial dot blot (“Euroimmun,” Lubeck, Germany, serum dilution 1:100). Therefore, a diagnosis of anti-Ma2 encephalitis was made. The patient was started on high-dose steroid therapy (6-methylprednisolone 1 g/day intravenous for 5 days). Since no clinical response was observed, intravenous immunoglobulins were administered (25 g/day for 5 days), though without any improvement. Her tumor continued to progress; therefore, she had palliative care and unfortunately died a few months later because of superimposed general complications.

## Discussion

We report on a patient with advanced lung cancer who developed acute-onset isolated nystagmus four months after starting treatment with anti-PD-L1 durvalumab. The patient tested positive for anti-Ma2 antibodies.

Recently, anti-Ma2-encephalitis has been described as a neurological immune-related adverse event (n-irAE) of ICIs [6, 12, 13]. ICIs are human IgG1 kappa monoclonal antibodies which activate the antitumor immunity [14]. However, the same upregulated immune response could cause a n-irAE through different mechanisms possibly related to a cross-reaction of activated T cells against central nervous system (CNS) self-antigens or cytotoxic and complement response against CNS self-components presenting ICIs target molecules (e.g., PD-L1) [4, 5].

In our patient, the tumor progressed, despite a combined approach including first-line chemotherapy (carboplatin and etoposide) and radiotherapy. Therefore, durvalumab therapy was started.

The acute onset of the neurological picture long after tumor diagnosis, its absence before treatment with ICI, along with its close temporal relationship with durvalumab, strongly addressed a diagnosis of a n-irAE associated with ICIs instead of a classical PNS [4, 6].

Ocular motor alterations have been previously reported both as paraneoplastic phenomena in anti-Ma2 encephalitis and as n-irAE triggered by ICIs. Notably, to our knowledge, this is the first reported case of torsional pendular nystagmus for both conditions.

Our patient’s nystagmus had a pendular waveform and a predominant conjugate torsional component. Etiology of acquired pendular nystagmus includes visual loss, demyelinating diseases [15–19], oculopalatal tremor (OPT) syndrome [20, 21], Whipple’s disease [22], hypoxic encephalopathy [23], serotonin syndrome [24], and sporadic cerebellar ataxia [25].

As previously reported for OPT syndrome, we can speculate that the pendular nystagmus could be caused by an altered error signal sent from the inferior olive to the cerebellar cortex, resulting in a disruption of motor learning [26]. Even though brain MRI did not show olivary hypertrophy, which can however take several months to develop, the disruption of the above-mentioned pathway could underly this peculiar type of nystagmus. This is also supported by the similarity between the pendular nystagmus in our case and that reported in a patient who suffered from an acute pontine hemorrhage involving the central tegmental tract, which is a common cause of OPT [27]. However, we can also speculate that the observed nystagmus could be explained by the disruption of other different pathways which are known to underly certain type of pendular nystagmus.

Indeed, the observation that patients affected by multiple sclerosis with pendular nystagmus have demyelinating lesions in the region of the paramedian tract cell groups [18] led to the hypothesis of an instability of the neural integrator that hold gaze position [28]. Such a mechanism could also explain the pendular torsional component seen in pendular seesaw nystagmus. In our patient, the superimposed disconjugate vertical eye movements could be interpreted as a seesaw component and could be thus explained by the emergence of a phylogenetically old response that should be aimed at maintaining the eyes aligned on the horizontal meridian during head tilt in lateral-eyed animals. This mechanism could also explain alternating skew deviation in patients with cerebellar lesions [29]. Therapeutic strategies usually adopted, though with discordant results, are represented by withdrawal of ICI associated with corticosteroids, as first line, and IV immunoglobulin, plasma exchange, rituximab, or cyclophosphamide, as second line-therapies [6].

Nystagmus and oscillopsia could be treated, as in OPT, with drugs which increase Purkinje cells GABAergic inhibition (i.e., clonazepam, alprazolam, primidone, and topiramate) or reduce their glutamatergic excitatory effects (i.e., memantine or topiramate) [26].

Despite discontinuing durvalumab and beginning immunotherapy [2, 5, 30–32], the abnormal eye movements of our patient did not improve, and she unfortunately died a few months later.

## Conclusion

Our report widens the clinical spectrum associated with anti-Ma2 encephalitis related to ICIs.

Since in recent years ICIs have become widely used therapeutic options, it is important that clinicians are aware of the rare but disabling neurological adverse events, as the isolated torsional pendular nystagmus reported,  which can  occur and pose both diagnostic and therapeutic challenges.

### Supplementary information

ESM 1 Video 1 Pendular nystagmus with a predominant torsional conjugate component and superimposed irregular vertical movement of the eyes with jerk and disconjugate components. (MOV 10308 kb)

ESM 2 Video 2 Video of patient’s nystagmus in left, right, and upward gaze. It was very difficult for the patient to maintain the lateral gaze position. Her nystagmus did not change in lateral gaze, while it became less intense when looking up. There was no gaze-evoked nystagmus or rebound nystagmus. (MP4 43872 kb)

## Data Availability

Data and videos used in the present paper are available from the corresponding author on reasonable request.
